# Tracking community outreach and engagement activities among National Cancer Institute-designated cancer centers

**DOI:** 10.1093/jnci/djae232

**Published:** 2024-09-23

**Authors:** Todd Burus, Caree R McAfee, Pamela C Hull, Amy E Leader, Christopher McNair

**Affiliations:** Community Impact Office, Markey Cancer Center, University of Kentucky, Lexington, KY, USA; Community Impact Office, Markey Cancer Center, University of Kentucky, Lexington, KY, USA; Community Impact Office, Markey Cancer Center, University of Kentucky, Lexington, KY, USA; Department of Behavioral Science, College of Medicine, University of Kentucky, Lexington, KY, USA; Sidney Kimmel Comprehensive Cancer Center, Thomas Jefferson University, Philadelphia, PA, USA; Sidney Kimmel Comprehensive Cancer Center, Thomas Jefferson University, Philadelphia, PA, USA

## Abstract

The National Cancer Institute’s (NCI) Cancer Center Support Grant mandates that NCI-designated cancer centers establish a Community Outreach and Engagement (COE) component to help direct efforts at reducing cancer burden within their catchment areas. Despite the critical role of COE offices, little is known about how they track and evaluate outreach activities and outcomes. We gathered information on current practices from representatives of 40 out of 65 COE offices using an online survey. Although nearly all responding centers (97.5%) tracked COE activities, no consensus existed on resources used, and satisfaction with current solutions was mixed (51.0% not satisfied). Respondents expressed need for a centralized, standardized, and comprehensive tracking solution to capture outreach events and external partnerships, automate report generation, and ensure alignment with COE aims. This study highlights challenges COE offices face with resource limitations and a heterogeneity of activities to track, as well as the need for a standard evaluation framework.

Starting in 2012, funding under the National Cancer Institute’s (NCI) Cancer Center Support Grant called for NCI-designated cancer centers to maintain a geographically defined catchment area in which they focus research and outreach to help reduce cancer burden (1,2). Current funding requirements include the establishment of a Community Outreach and Engagement (COE) component that engages community partners to inform research priorities, facilitate clinical trial accruals, and direct cancer prevention and control efforts within the catchment area (3). Reporting progress on these goals requires accurately tracking relevant COE activities and outcomes; however, the mechanisms by which individual cancer centers currently handle such tracking needs is unknown.

We prepared an 8-question online anonymous survey in REDCap for the purposes of 1) capturing information about current resources being used to track COE activities among US cancer centers, and 2) gathering opinions about the sufficiency of current solutions and what ideal solutions should include (Supplementary Methods, available online). The survey was distributed across the American Association of Cancer Institutes (AACI) COE email Listserv on May 3 and July 8, 2024. This Listserv consists of leadership and staff in COE offices across AACI-affiliated institutions. All Listserv subscribers had access to submitted responses, and participation was voluntary. Analysis was restricted to respondents from NCI-designated cancer centers. The University of Kentucky Institutional Review Board determined this study to be for quality improvement and thus not human subject research.

We received 58 responses from individuals at 45 different cancer centers. Seven responses from 5 centers were excluded due to not being affiliated with an NCI-designated cancer center. Among respondents from the 40 remaining centers (representing 61.5% of NCI-designated cancer centers required to have a COE component), all but 1 center (97.5%) indicated that they were currently tracking COE activities in some way. Twenty-one individual responses (41.2%) were from associate or assistant directors (AD) of COE, 20 (39.2%) were from other administrative staff (eg, directors of operations, program managers/coordinators, ADs of non-COE departments), 4 (7.8%) were from researchers/analysts, and 6 (11.8%) were from other staff (Table 1).

**Table 1.** Satisfaction with current community outreach and engagement (COE) activity tracking solution by role

Role	n	Satisfied with current solution (%)
AD COE	21	12 (57.1%)
Administration	20	10 (50.0%)
Researcher/analyst	4	2 (50.0%)
Other Staff	6	1 (16.7%)
Total	51	25 (49.0%)

AD = associate or assistant director.

A majority of cancer centers indicated that they use Microsoft Excel/Google Sheets (70.0%) and REDCap (62.5%) to assist in activity tracking (multiple responses were allowed), with other resources being used to a lesser extent (Table 2). Just over half of individuals (51.0%) said they were not satisfied with their center’s current activity tracking solution, including 5 centers where multiple respondents had differing opinions. ADs expressed greater satisfaction with current tracking solutions (57.9%) than the staff members typically tasked with inputting data (16.7%). Several respondents using REDCap noted that it is “user friendly,” “flexible,” and “customizable,” although some remarked that it is “not perfect.” Similar comments were given with respect to Excel. Respondents shared that an ideal solution would be “centralized,” “standardized,” and “comprehensive,” with the ability to “capture partnerships” as well as activities. Some respondents indicated they would like a solution that incorporates some sort of automation, particularly as pertains to report generation.

**Table 2.** Resources used by cancer centers to track community outreach and engagement activities **(**responses reflect use among 40 NCI-designated cancer centers represented in survey; centers may have reported using more than one resource)

Resource used	n (%)
Microsoft Excel or Google Sheets	28 (70.0%)
REDCap	25 (62.5%)
Qualtrics	9 (22.5%)
Online Survey Software	1 (2.5%)
Google Forms	5 (12.5%)
Microsoft Access	0 (0.0%)
Custom Software Solution	2 (5.0%)
Other^a^	12 (30.0%)

a Other resources reported: hospital EMR (2), Salesforce (2), Smartsheet (2), DOMO (1), Infoready (1), Partnership Tracking (1), Survey123 (1), and Microsoft Word (1).

When asked what was most important for them to track, a majority of respondents cited outreach events (80.4%) and community partnerships (54.9%). The need to record detailed information about these interactions was expressed, both of a singular activity and over time. Some respondents commented on tracking information and impact in alignment with the specific aims of COE, although what this entailed was not entirely clear. Some referred to “aims” broadly, whereas others enumerated between 2 and 4 different aims of interest (eg, “surveillance activities,” “cancer education and outreach,” “engagement,” “research interactions”). As one AD of COE remarked about tracking needs for their institution, “by far, the most important is impact (however defined).”

The lack of a standard framework and tools for tracking and evaluating activities has led to a fractured approach being taken across cancer centers, as demonstrated in this survey. Our findings highlight some of the common challenges facing COE offices when it comes to tracking activities and impact. One major challenge is the heterogeneity of elements across COE activities (4). For example, capturing the reach and impact of a cancer screening event is different from documenting the evolution of a community partnership. Such differences require different tools (or, at minimum, different inputs within the same tool) to record these elements. Given that many cancer centers are conducting similar activities, and some cancer centers even have overlapping catchment areas, establishing a common set of core measures for tracking would increase efficiency (5). Another challenge is the ever-present issue of limited resources. This can show up in budgetary constraints on purchasing commercial tracking solutions, a shortage of skilled personnel to design and maintain tracking solutions, or an excess burden on outreach personnel to record activities and generate reports. Lastly, evaluating the impact of COE is central to enhancing the reach and sustainability of COE activities and fulfilling the mission of cancer centers to reduce cancer burden in their catchment area. Documenting this impact is necessary to show that COE offices are meeting the aims required for NCI designation. The lack of a standard set of metrics for reporting impact creates a challenge in that each center is left to decide for themselves how to best communicate the importance of what they are doing, both internally and externally (6). Reducing the variability in evaluation, at least among a core set of common metrics, would allow centers to design more efficient tracking solutions to capture the necessary elements (7).

Just as collaborative, open-source resources such as Bioconductor (a platform for bioinformatics packages) (8) and Cancer InFocus (a data gathering and visualization platform for cancer surveillance) (9) have benefited individuals in other parts of the cancer center, the same approach would likely prove useful for COE activity tracking solutions as well (10). Intentionally designing an end-to-end platform for cancer centers that can be freely distributed would create greater efficiency and standardization—traits many respondents indicated as being most needed. Such a platform would simplify the task of evaluation and report generation, create less overhead for resource development, and allow COE staff to plan activities with clear goals already in mind. Given that a majority of centers in our survey reported using free REDCap software to assist in their tracking needs, this could be an ideal software to consider as the starting point for such an effort. Ultimately, no one solution will be able to meet all the needs of every cancer center, but it is possible to envision a resource that meets the core needs, establishing a common set of data elements that all cancer centers could use, while allowing for further customization as desired.

With regard to implementation, existing connection points for COE—such as the annual Cancer Center Community Impact Forum and the AACI COE Listserv—could be used as focal points to spark collaboration and advance conversations around core metrics. Building on the collected wisdom of COE leaders from multiple institutions and diverse backgrounds would help inform a process that answers the questions frequently brought up in external advisory board meetings and Cancer Center Support Grant site visits, while also considering the staffing limitations that exist. Leveraging insights from past collective efforts such as the NCI’s Cancer Information Service, in terms of collecting evaluation data across sites and working toward a common research agenda, is also important (11). Although the guidelines for COE in the Cancer Center Support Grant have been refined over time, certain criteria for evaluation remain ambiguous (6,12). Together, community-engaged researchers and staff within COE could take charge to define and implement among themselves what the standards for evaluating COE activities look like.

Establishing offices of COE to direct outreach within the catchment area was an important step toward helping cancer centers reduce the cancer burden. To maximize these efforts, however, we must address the lack of consensus that currently exists surrounding the tools and processes used to track and evaluate activities.

## Supplementary Material

djae232_Supplementary_Data

## Data Availability

All data underlying this study are available from the corresponding author by request.
