# Supplementary material for: Tracking community outreach and engagement activities among National Cancer Institute-designated cancer centers
Source: J Natl Cancer Inst. 2024 Sep 23;117(2):335–7. doi: 10.1093/jnci/djae232 (PMC11807429; doi:10.1093/jnci/djae232)
Supplement: djae232_Supplementary_Data [file djae232_supplementary_data.pdf]

Supplementary Materials

Supplementary Methods: Cancer Center COE Tracking Tools Survey

The intention of this survey is to understand the current landscape with respect to Community Outreach and Engagement tracking tools currently in use.

The data collected in this survey will be used to determine the following:

- Whether there is a need to build a standardized, shareable tool to decreased the barriers for COE teams to track activities, and
- If there are trends within tools currently used or in development worth having follow up conversations on

Depending on the results of this survey, more specific information may be requested in the future.

Thank you in advance for your time!

---

1. Institution/Affiliation: ___________________

2. Role/Title: ___________________

3. Does your institution currently track Community Outreach and Engagement (COE) Activities?
   a. Yes
   b. No

4. [If "Yes" on #3] What tool(s) are used to track these activities? (*multiple choice*)
   a. Microsoft Excel/Google Sheets
   b. REDCap
   c. Qualtrics
   d. Online Survey Software (e.g. Jotform, Survey Monkey, etc.)
   e. Google Forms
   f. Microsoft Access
   g. Custom Software Solution
   h. Other

5. [If checked "Other" on #4] Please describe: ___________________

6. [If "Yes" on #3] Are you satisfied with your current solution?
   a. Yes
   b. No

7. [If "Yes" on #3] What is most useful about your current solution? (*open ended*)

8. Please describe what an ideal solution would look like. (*open ended*)

9. In your experience, what types of information are most important to track for COE groups? For example: cancer screening events, community outreach partners, etc. (*open ended*)
